# Autonomy in socially assistive robotics: a systematic review

**DOI:** 10.3389/frobt.2025.1586473

**Published:** 2025-09-19

**Authors:** Romain Maure, Barbara Bruno

**Affiliations:** Socially Assistive Robotics with Artificial Intelligence Lab, Karlsruhe Institute of Technology, Karlsruhe, Germany

**Keywords:** human-robot interaction, socially assistive robotics, level of autonomy, artificial intelligence, robot teleoperation, Wizard of Oz, transparency, systematic review

## Abstract

Socially assistive robots are increasingly being researched and deployed in various domains such as education, healthcare, service, and even as collaborators in a variety of other workplaces. Similarly, SARs are also expected to interact in a socially acceptable manner with a wide audience, ranging from preschool children to the elderly. This diversity of application domains and target populations raises technical and social challenges that are yet to be overcome. While earlier works relied on the Wizard-of-Oz (WoZ) paradigm to give an illusion of interactivity and intelligence, a transition toward more autonomous robots can be observed. In this article, we present a systematic review, following the PRISMA method, of the last 5 years of Socially Assistive Robotics research, centered around SARs’ level of autonomy with a stronger focus on fully and semi-autonomous robots than non-autonomous ones. Specifically, to analyse SARs’ level of autonomy, the review identifies which sensing and actuation capabilities of SARs are typically automated and which ones are not, and how these capabilities are automated, with the aim of identifying potential gaps to be explored in future research. The review further explores whether SARs’ level of autonomy and capabilities are transparently communicated to the diverse target audiences above described and discusses the potential benefits and drawbacks of such transparency. Finally, with the aim of providing a more holistic view of SARs’ characteristics and application domains, the review also reports the embodiment and commonly envisioned role of SARs, as well as their interventions’ size, length and environment.

## 1 Introduction

Socially Assistive Robotics (SAR) describes a class of robots that stand at the intersection of assistive robotics, which includes robots that provide assistance to a user, and socially interactive robotics, which includes robots that communicate with a user through social interaction (Feil-Seifer and Matarić, 2011). As assistive robots, SARs’ core function is to provide assistance in a variety of domains ranging from education to health or tasks requiring collaborative teamwork (Breazeal et al., 2016). As social robots, SARs are expected to interact in a natural manner with people of different ages, expertise and cultures (Breazeal et al., 2016). To successfully interact with and properly assist users, SARs require to be equipped with appropriate interaction and actuation capabilities. Similarly, SARs also need to be equipped with sensing capabilities to be able to sense their environment and react appropriately to humans’ inputs and actions. Creating social robots that are competent and capable assistants for people raises technical challenges that are yet to be overcome (Breazeal et al., 2016). As a result of these challenges, many SAR applications rely on the Wizard-of-Oz (WoZ) paradigm, which refers to a person (usually the experimenter and often hidden to the target user) remotely operating a robot and controlling some or all of the robot’s sensing and actuation capabilities, such as its vision, hearing, navigation, speech, gestures, etc (Riek, 2012). WoZ may involve any amount of control along the autonomy spectrum, from full autonomy to full human control (or no autonomy), with human-robot shared autonomy (or semi-autonomy) anywhere in between (Riek, 2012). While technical challenges are often a motivation to choose no or shared autonomy over full autonomy, other factors can also enter into consideration. For example, when robots are used for highly critical tasks that have potential for human safety concerns, less autonomous robots can be preferred (Elbeleidy et al., 2022). Similarly, when robots are used in tasks which require a clear chain of accountability, less autonomous robots can also be recommended so that blame can be appropriately attributed (Elbeleidy et al., 2022). Finally, teleoperation can also be preferred in applications in which the operator is the target user itself and operating the robot is part of the SAR intervention. Although WoZ has advantages and is a convenient tool for human-robot interaction (HRI) researchers, many raise concerns about this technique. For example, Clabaugh and Matarić (2019) consider WoZ methods intractable in SAR domains that require long-term and real-world interventions. As described in Riek (2012), many researchers argue that WoZ has methodological flaws, to the point of stating that there is no real human-robot interaction when using this technique, but rather a human-human interaction through the proxy of a robot. Additionally, Riek (2012) also mentions that researchers are concerned about the ethical flaws of this method, both for the target user, who is subject to deception, and for the operator, who is required to perform deception.

The above discussion highlights how the topic of autonomy in SARs is as old as SARs themselves, and still far from being solved. Indeed, we identified three reviews that explore the relation between SARs and their level of autonomy (LoA).

Clabaugh and Matarić (2019) focus exclusively on fully autonomous robots and explore how full autonomy is achieved. They analyse full autonomy along two dimensions. The first dimension corresponds to the interaction complexity, which is defined by the intervention’s group size and length, as well as the robot’s embodiment and role. The second dimension corresponds to the computational complexity and intelligence of the SAR system, which is defined by the environment’s observability and discreteness, as well as the number of percepts (i.e., sensing modalities) and the level of reasoning of the robot system employed. Riek (2012) focuses solely on studies using the WoZ paradigm (non- and semi-autonomous robots). The review explores how the WoZ technique is typically employed in HRI by classifying studies using criteria proposed by different authors (Fraser and Gilbert, 1991; Green et al., 2004; Steinfeld et al., 2009; Kelley, 1984). These criteria evaluate, for example, the type of WoZ model used (Wizard of Oz, Wizard with Oz, Wizard and Oz, Oz with Wizard, Oz of Wizard, Wizard nor Oz) (Steinfeld et al., 2009), the possibility to simulate the system in an autonomous way in the future (Fraser and Gilbert, 1991), the necessity of providing training to wizards (Fraser and Gilbert, 1991), whether instructions were given to the target users to specify what they could do during the interaction (Green et al., 2004), whether the use of the WoZ technique is part of an iterative design process or not (Kelley, 1984), *etc.* Finally, Elbeleidy et al. (2022) explores the whole spectrum of autonomy (from non-autonomous to fully autonomous). The authors use Beer et al. (2014)’s definition of autonomy: “The extent to which a robot can sense its environment, plan based on that environment, and act upon that environment with the intent of reaching some task-specific goal without external control.” Using this definition, they compare researchers’ choices of robot’s level of autonomy with frameworks providing guidelines on how to select a robot’s level of autonomy in accordance to the type of intervention and users (Beer et al., 2014), and suggest that there is a mismatch between the two. They also demonstrate that researchers rarely provide a rationale concerning their choice for the robot’s level of autonomy. While Elbeleidy et al. (2022) shed light on an interesting gap in the literature, they only consider the overall level of autonomy of social robots, with no insights on the underlying technologies, unlike the reviews of Clabaugh and Matarić (2019) and Riek (2012).

In the present review, we employ the same search pattern as the one used by Clabaugh and Matarić (2019) (further described in Section 2). By doing so, we aim to skew our research towards publications whose SAR systems presents some degree of autonomy, with the overarching goal of understanding how and through which means autonomy is achieved in HRI. As opposed to the review of Clabaugh and Matarić (2019), however, we do not exclude semi- and non-autonomous SAR works that would result from the search. By doing so, we aim to understand which sensing and actuation capabilities are typically automated, and which ones are not, and attempt to provide insights concerning the choice of robots’ level of autonomy at the functionality level, thus potentially gaping the lack of researcher’s rationale identified by Elbeleidy et al. (2022). It should be noted that, while the search pattern allows to identify publications whose SAR system presents some degree of autonomy (as stated by Clabaugh and Matarić (2019): “The terms in the query only enforce that a paper includes some form of social HRI and ML or AI for automation”), the search pattern does not allow to provide a comprehensive view of non-autonomous SARs. To summarize, this article contributes to the current state of research in Socially Assistive Robotics by providing an up-to-date systematic review focusing on the level of autonomy employed in SAR applications. We report the robots’ embodiment and commonly envisioned role, as well as the interventions’ size, length and environments of SARs. We study autonomy by analysing which sensing and actuation capabilities of social robots are typically automated, and which ones are not, and how these capabilities are automated. Finally, we also study the level of transparency concerning robots’ autonomy and capabilities.

The article is structured as follows. First, we present the methodology of the survey in Section 2. Following the coding scheme introduced in the survey methodology, we analyse the included publications in Section 3 and discuss the insights emerging from the analysis in Section 4. Finally, we conclude and provide opportunities for future research in Section 5.

## 2 Materials and methods

### 2.1 Identification, screening and selection of relevant publications

The present study is a systematic review following the PRISMA approach (Page et al., 2021). We used Google Scholar to identify records and limited the search over the last 5 years (from January 2019 to July 2023). We limited our search to the same venues as the ones indicated in Clabaugh and Matarić (2019): the International Conference on Human-Robot Interaction (HRI), the International Conference on Robot and Human Interactive Communication (RO-MAN), the Conference on Human Factors in Computing Systems (CHI), the Interaction Design and Children Conference (IDC), the International Conference on Robotics and Automation (ICRA), the International Conference on Intelligent Robots and Systems (IROS), the Robotics: Science and Systems conference (RSS), the International Conference on Development and Learning and Epigenetic Robotics (ICDL-EpiRob), the Conference on Robot Learning (CoRL), the AAAI Conference on Artificial Intelligence (AAAI), the International Conference on Multimodal Interaction (ICMI), the International Conference on Machine Learning (ICML), and the Conference on Neural Information Processing Systems (NeurIPS). Similarly, and as indicated in Section 1, we used the same search pattern as the one used by Clabaugh and Matarić (2019):

(social OR sociable OR socially) AND (“machine learning” OR “artificial intelligence”) AND “human-robot interaction.”

The search returned 1,138 records. Among them, 10 were identified as duplicates and removed and 37 were removed because they were published at venues excluded from our search. Among the 1,091 remaining reports, one could not be retrieved because it was not correctly referenced anymore, leaving 1,090 articles to be assessed for eligibility. We excluded 51 reports for being meta-analyses. Specifically, these articles do not present a primary study but rather focus on synthesizing, comparing and discussing the work described in other studies. Similarly, we further excluded 185 reports due to their type. These articles are workshop papers, extended abstracts, student competition papers, video demonstration papers, position papers, summaries of a person’s work (e.g., full PhD theses) or project plans. Lastly, we excluded 784 reports as out-of-scope for this review. In particular, an article was considered out-of-scope if it fell in one of the following cases:

1. The work lacks a clear socially assistive application.
2. The work lacks details about the robot’s approach to sensing and acting.
3. The work was covered and expanded on in a subsequent publication.
4. The work lacks a human-robot interaction:
  - There is no interaction between a human and a robot.
  - Human participants only interact with a virtual robot (AR/VR or agent on a display).
  - Human participants only interact with a physical robot in an online format (videoconference).
  - Human participants only watch a video of a pre-recorded human-robot interaction.

**FIGURE 1:**
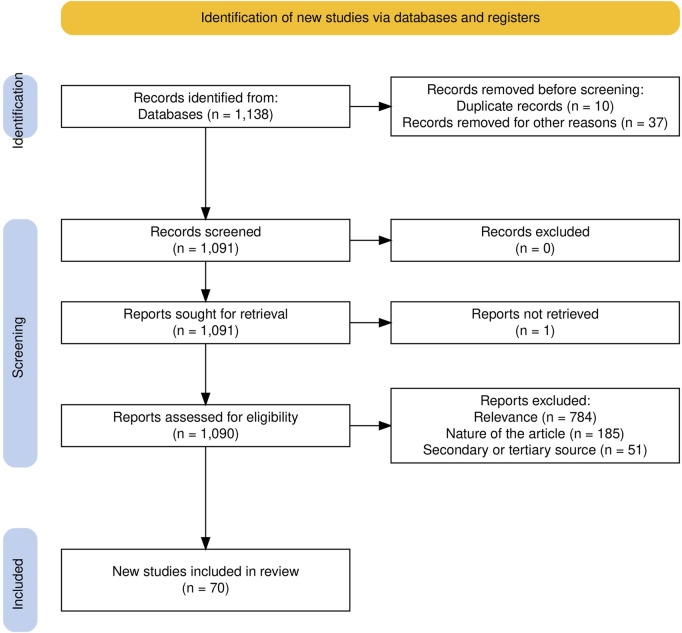
PRISMA flow diagram for the stages of identification, screening and inclusion.

**FIGURE 2:**
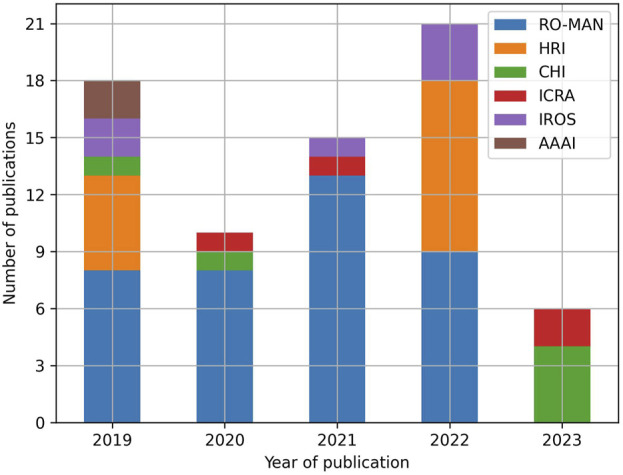
Number of publications by venue and by year.

In the end, 70 studies were included in the review. Figure 1 summarizes the flow of information through the stages of identification, screening, and inclusion. Figure 2 presents the distribution of publications by venue and by year. Please notice that the low number of publications in 2023 is partly due to the fact that this review only considers articles published in the first half of 2023.

### 2.2 Coding of the included publications

After the stages of identification, screening and selection of relevant publications, we coded each of the included 70 publications using the coding scheme^1^ described below. As introduced in Section 1, and to the best of our knowledge, there is currently no unique and standardized way of evaluating SAR autonomy. The coding scheme of the present review is partially inspired by the coding scheme described in Clabaugh and Matarić (2019). However, since we do not only consider fully autonomous systems, as done in Clabaugh and Matarić (2019), but also non- and semi-autonomous systems, some elements of Clabaugh and Matarić (2019)’s coding scheme were adapted or discarded, while new elements were added, to fit our specific goals and research interests.

**FIGURE 3:**
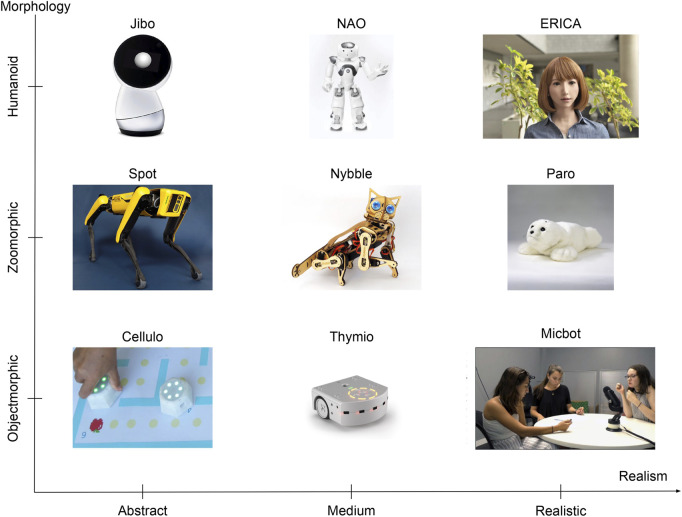
Classification of robots based on their morphology and their realism. The Micbot image is taken from (Tennent et al., 2019).

- **Field of application** reports the domain for which the robotic intervention is intended. The publications are classified into the following domains: health, education and workplace.
- **Subfield of application** provides further details on the robotic application’s domain. Examples of subfields for the health domain are physical activity, body hygiene or mental wellbeing, among others.
- **Age** is classified using Erikson’s stages of development (Erikson and Erikson, 1998): infancy (0–1 years old), early childhood (1–3 years old), preschool (3–6 years old), school-age (6–12 years old), adolescence (12–19 years old), early adulthood (19–25 years old), adulthood (25–64 years old), elderly (65+ years old). We use the age range of the participants’ population to determine the corresponding Erikson’s stages of development. As a result, a publication may include multiple Erikson’s stages of development. In the cases of publications that only specify the mean μ and standard deviation σ of the participant’s population’s age, we determine the Erikson’s stages of development using an age range equal to μ±σ . In the cases of publications that only specify the mean age of the population, we infer the Erikson’s stage of development by looking at the stage in which the mean falls into. Publications that do not report the age of their population are classified as unspecified.
- **Robot’s name** reports the name of the robot used in the study.
- **Robot’s morphology** comprises three classes: humanoid, zoomorphic and objectmorphic. Robots are classified as humanoid, zoomorphic or objectmorphic if their morphology mainly resembles, more or less realistically, the one of a human, an animal or an object respectively. Figure 3 provides examples of robots classified as humanoid [Jibo, NAO and ERICA (Glas et al., 2016)], examples of robots classified as zoomorphic [Spot, Nybble and Paro (Shibata and Wada, 2011)], as well as examples of robots classified as objectmorphic [Cellulo (Özgür et al., 2017), Thymio (Mondada et al., 2017) and Micbot (Tennent et al., 2019)].
- **Robot’s realism** is assessed on a three-point ordinal scale as: abstract, medium and realistic (Figure 3). A robot is considered abstract if it exhibits only a few, fundamental characteristics and morphology of a human, animal, or object. For example, Jibo is considered abstract, as it solely possesses a torso, a head, and a vertical posture similar to that of a human. Similarly, Spot is considered abstract due to its overall quadrupedal animal shape, which however does not correspond to any specific animal. Conversely, robots are considered realistic if they display a sufficient number of characteristics of the reference morphology. For instance, ERICA is equipped with synthetic skin and hair, has human-like facial and body features, and wears human clothing, rendering it very similar to a human. Similarly, Paro not only has the morphology of a baby seal, but it is also covered in synthetic fur, making it more easily recognizable.
- **Robot’s role** is divided into four classes: subordinate, peer, superior/tutor and multiple roles. A robot is considered to have multiple roles if it enacts a different role in different conditions (Salomons et al., 2022a), or in accordance with the user’s preferences or actions (Domínguez-Vidal et al., 2021).
- **Intervention size** is assessed on a five-point ordinal scale as: dyadic (size of 2), triadic (size of 3), small group (size of 4–9), medium group (size of 10–14) and large group (size of 15+). The robot is included in the group’s size count. Conversely, people who relate to the intervention but are not members of the target population are not: for example, in the context of an educational robot, an adult teleoperating the robot such as it is done in Vogt et al. (2019) is excluded from the group size count.
- **Intervention length** is assessed on a five-point ordinal scale as: single session, 2-6 sessions, 1–3 weeks, 1–11 months, more than 1 year. Note that this rating does not take into account the time between sessions: a study composed of five sessions spread over a month would fall in the 2-6 sessions category, for example, exactly as one in which the five sessions took place over the span of a few days, with multiple sessions per day. This rating also does not account for the duration of each individual session: a study such as Salomons et al. (2022b), composed of 15–25 min interventions, repeated each day over 14 days, would fall in the 1–3 weeks category, for example. We also only focus on the duration of the SAR intervention in itself: a final evaluation session is, for example, discarded from the count.
- **Intervention environment** reports the environment in which the user study has been conducted. Examples of intervention environments are school/university, home, clinical environment and research laboratory. Publications that do not report the intervention environment are classified as unspecified.
- **Sensing** reports who is taking care of sensing the environment, on behalf of the robot, during the interaction. We identify three possible classes: human (if a human is responsible of the robot’s perception), robot system (if the robot is provided with perceptual information collected by on-board sensors or external hardware components), and unused (no sensing is done or mentioned). We report this information for each of the following sensing modalities: vision, hearing, touch, and other. Note that our assessment exclusively focuses on the intervention itself: any sensing modality used to collect data solely for offline post-processing is not considered. Furthermore, some works rely on a combination of human and robot sensing for a same sensing modality. For example, in Mizumaru et al. (2019), vision is handled both by the robot and a human operator. We code such cases as human sensing, as the robot is reliant (albeit partly) on human input. Conversely, other publications present a robotic intervention in which a same sensing modality can be managed by either a human or the robot, autonomously. For example, in Nakanishi et al. (2021), vision can be handled by a human operator, or autonomously by the robot. Such cases are coded as robot sensing, given that the robot is able to autonomously detect and process information along that sensing modality, and the possibility of human involvement is just an optional feature. Finally, in case a publication lacked clarity with respect to sensing, it would be excluded according to the exclusion criteria “The work lacked details about the robot’s approach to sensing and acting.” For instance, if the authors of a publication mention the use of a sensing modality, but it is unclear whether this sensing modality is used solely for data collection and offline post-processing purposes or it is actively used during the intervention, the publication would be excluded. Similarly, if the authors of a publication mention the use of a sensing modality, but it is unclear whether this sensing modality is handled autonomously by the robot or by a human operator, the publication would be excluded. To perform the coding of the sensing modalities and identify whether a publication provides sufficient details, a two-pass method was used. In the first pass, the coder would go through the article adopting a fast reading approach^2^, to identify all sensing modalities mentioned in the paper. During this pass, the coder would search for any term that could generally be associated with any of the considered sensing modalities (e.g., for the hearing modality, the coder would look for terms such as “microphone,” “speech recognition,” “audio recording,” etc.). Please notice that no specific set of keywords was systematically used to this purpose, also to take into account the possibly different linguistic conventions of different sub-communities and linguistic proficiency levels). If a considered sensing modality was not mentioned in the article, it would be marked as “unused,” as described above. For each considered sensing modality mentioned in the article, the coder would identify all the sections of the paper that refer to it and read them with the aim of identifying who handles it (i.e., a human or the robot system, as described above). If the answer could be found, the coder would mark the answer in the coding sheet and move on to the next mentioned sensing modality, or coding item if no other considered sensing modality is left. If no clear answer could be found, the coder would perform a second pass, in which the paper is thoroughly read in full. In case the coder could still not confidently identify how the sensing modality is handled after the second pass, the article would be excluded on the grounds of a lack of sufficient details about the robot’s approach to sensing or acting. Notice how this conservative approach aims at maximizing the precision of the analysis, at the expense of possibly excluding articles that are clear in all parts except for the handling of even just one considered sensing modality.
- **Actuation** reports who decides how the robot should act during the interaction. We again consider three classes: human, robot system, and unused (no acting is done or mentioned). We report this information for each of the following action modalities: movement, speech/sound, facial expression, lights, and display. Note that we focus on the action level and not on a goal level: a robot can be considered fully autonomous, even if it has been tasked to perform a specific task by a human, as long as each action to accomplish this task is performed autonomously by the robot. For example, in Kraus et al. (2022), human participants were able to give high level goals to a robot such as fetching food, or cleaning the environment. Even though the tasks to be accomplished were decided by a human, the robot was classified as fully autonomous, because all of the action modalities used to carry out each task were handled in a fully autonomous way by the robot. Note that we only consider modalities for direct (inter)action on the target population: a display used to convey information to an operator is not considered, unless the operator is a member of the target population. Additionally, displays used as a robot’s face (such as the face of Jibo) are classified as part of the facial expression category and not the display category. Finally, the same reasoning described in the sensing section above applies for the cases where an action modality is jointly handled by a human and the robot, or either by a human or the robot. The same reasoning as the one described above also applies when it comes to a lack of clarity with respect to actuation modalities. If the authors of a publication mention the use of an actuation modality, but it is unclear whether this modality is handled autonomously by the robot, or manually handled by a human operator, the publication would be excluded. The same two-pass process described above for the sensing modalities was used to code the actuation modalities and identify whether an article should be excluded from the survey due to a lack of details about the considered action modalities.
- **Level of autonomy** is divided into three classes: non-autonomous, semi-autonomous, and fully autonomous. The level of autonomy is decided on the basis of the sensing and actuation items described above. A robot is classified as non-autonomous if its sensing and actuation capabilities are only human-based. A robot is classified as semi-autonomous if at least one of its sensing or actuation capabilities is handled by a human operator and at least one of its sensing or actuation capabilities is handled by the robot system. Finally, a robot is classified as fully autonomous if all of its sensing and actuation capabilities are handled by the robot system. It is important to note that, according to this definition, a SAR system does not necessarily need to rely on artificial intelligence to be classified as fully autonomous: a SAR system solely relying on handcrafted behaviours with no need for human intervention during the interaction, would in fact also be classified as fully autonomous. Please also note that dynamic shared autonomy is beyond the scope of this review. As previously described, sensing or actuation abilities that are handled both by a robot and a human operator are classified as human sensing/actuation, given that the robot is reliant (albeit partially) on human input. Additionally, sensing or actuation abilities that can be handled either by a human operator or autonomously by the robot are classified as robot sensing/actuation, given the fact that human operation is an optional but not necessary feature. This choice to classify autonomy in a static way is made because our main research interest lies in identifying gaps in SAR autonomy at the functionality level, rather than exploring cases of shared autonomy and how such cases are typically handled.
- **Operator** reports who is the operator in the case of non-autonomous and semi-autonomous robots. Examples of operators are researchers, caregivers, target users themselves, *etc.*
- **Transparency on the level of autonomy** reports if the level of autonomy is disclosed, prior to the SAR intervention, to the target user(s) or not. We report this information only for the publications employing a semi-autonomous or non-autonomous robot.
- **Transparency on the robot’s capabilities** reports if the robot’s capabilities (in terms of sensing and actuation) are explicitly disclosed, implicitly disclosed, or not disclosed, prior to the SAR intervention, to the target user(s).

## 3 Results

### 3.1 Fields of application

**FIGURE 4:**
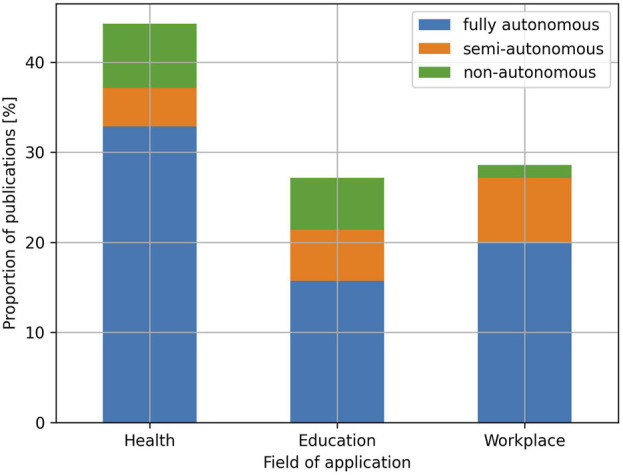
Proportion of publications per field of application and level of autonomy.

Concerning the fields of application of SARs, 44.3% of the publications included in this review focus on the health domain, while 27.1% employ social robots for applications related to the field of education, and 28.6% explore the use of social robots in the workplace (Figure 4). Among the health domain, the applications are diverse: helping individuals with ASD (Ramnauth et al., 2022; Carpio et al., 2019; Hijaz et al., 2021), visual impairment (Antunes et al., 2022; Kayukawa et al., 2023) or auditory impairment (Uluer et al., 2020; Chang et al., 2022), promoting physical activity (Kothig et al., 2021; Cooney et al., 2020; Salomons et al., 2022b; Cao et al., 2022; Li X. et al., 2023) or mental wellbeing (Farrall et al., 2023; Dino et al., 2019; Zhang et al., 2023), providing assistance on feeding (Gallenberger et al., 2019; Shaikewitz et al., 2023) or body hygiene (Palinko et al., 2021; Unnikrishnan et al., 2019; Ye et al., 2022), delivering services in clinical environments (Odabasi et al., 2022; Horn et al., 2022) or acting as a telepresence device for caregivers (Fiorini et al., 2020) or patients (Mackey et al., 2022; Wang et al., 2021). In the field of education, the disciplines of science, technology, engineering and mathematics (STEM) are the most commonly considered (Nasir et al., 2019; Donnermann et al., 2020; Ramachandran et al., 2019; Charisi et al., 2021), followed by language learning (Vogt et al., 2019; Kim et al., 2019), storytelling (Zhao and McEwen, 2022; Park et al., 2019), and motor development (Tozadore et al., 2022; Kouvoutsakis et al., 2022), among others. Finally, similarly to the health domain, the use of SARs in the workplace is represented by a variety of scenarios, including as receptionists (Mishra et al., 2019; Hwang et al., 2020; Gunson et al., 2022), waiters (McQuillin et al., 2022; Naik et al., 2021), tour-guides (Del Duchetto et al., 2019; Cauchard et al., 2019), assembly workers (Rajavenkatanarayanan et al., 2020; Lambrecht and Nimpsch, 2019), service providers in retail environments (Takada et al., 2021; Lewandowski et al., 2020), cooking assistants (Yamamoto et al., 2021) or video recording assistants (Li J. et al., 2023).

### 3.2 Populations

**FIGURE 5:**
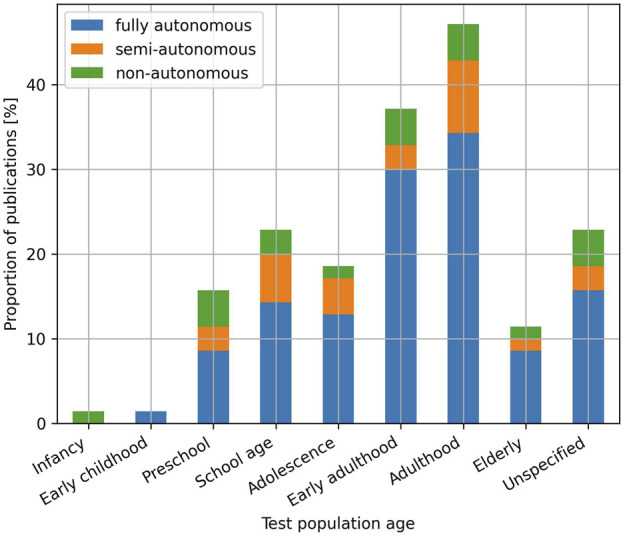
Proportion of publications per age category and level of autonomy. Please notice that a publication can fall into multiple age categories, as explained in Section 2.2.

Concerning the populations recruited to test the robotics applications (Figure 5), the most represented age categories are the early adulthood (Salomons et al., 2022b; Donnermann et al., 2020) and adulthood categories (Kayukawa et al., 2023; Takada et al., 2021), which are included in respectively 37.1% and 47.1% of the publications analysed in this review. The widespread presence of these age categories is likely explained by the ease to recruit and conduct experiments with such populations. Following these categories, the preschool (Vogt et al., 2019; Tolksdorf and Rohlfing, 2020), school age (Antunes et al., 2022; Kim et al., 2019) and adolescence (Azizi et al., 2022; Nasir et al., 2019) are included in respectively 15.7%, 22.9% and 18.6% of the publications. Most of these age categories are represented by the education domain. Finally, the least represented populations, which are also the most vulnerable, are the elderly (Luperto et al., 2019; Fiorini et al., 2020), early childhood (Zhao and McEwen, 2022) and infancy populations (Kouvoutsakis et al., 2022), which are present in respectively 11.4%, 1.4% and 1.4% of the studies. In a Kendall’s tau-b correlation analysis investigating the relationship between the age of the populations (excluding the unspecified category) and their representation in the publications included in this review, a strong and nearly statistically significant positive correlation was observed, $\tau_{b}=0.545,\;p=0.061$ .

### 3.3 Robots used

**FIGURE 6:**
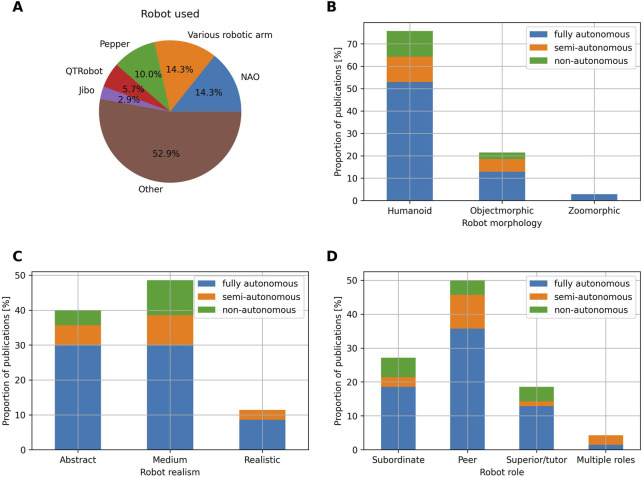
**(A)** Proportion of the robots used in the studies included in the review. **(B)** Proportion of publications per robot morphology and level of autonomy. **(C)** Proportion of publications per robot realism and level of autonomy. **(D)** Proportion of publications per robot role and level of autonomy.

Concerning the robots used in the studies, NAO, Pepper (Pandey and Gelin, 2018), QTRobot and Jibo are the most commonly employed and represent respectively 14.3%, 10%, 5.7% and 2.9% of the publications (Figure 6A). Various robotic arms such as UR5, KUKA LBR iiwa or FRANKA EMIKA PANDA are also used and collectively account for 14.3% of the publications (Salomons et al., 2022a; Li J. et al., 2023; Ye et al., 2022; Kowalski et al., 2022). The figure also shows that the majority of the studies are conducted with a plethora of diverse robots (52.9%), some being commercially available such as Turtlebot, Double or TIAGo, while some are *ad hoc* designed, such as Micbot (Tennent et al., 2019), POP Cart (Takada et al., 2021), RIMEPHAS (Palinko et al., 2021) or Cobbie (Lin et al., 2020).

Most of the robots used have a humanoid morphology (75.7%), while objectmorphic robots represent 21.4% of the publications and zoomorphic robots only represent 2.9% of the publications (Figure 6B). Objectmorphic robots take various forms: a suitcase (Kayukawa et al., 2023), a backpack (Cao et al., 2022), a microphone (Tennent et al., 2019), a wheeled vehicule (Domínguez-Vidal et al., 2021) or a drone (Cauchard et al., 2019), among others. Zoomorphic robots are only employed in two studies: one uses the Luka robot (owl-shaped robot) in a storytelling activity for children (Zhao and McEwen, 2022), while the other used the Keepon robot (canary-shaped robot) in an interaction promoting physical activity in early adulthood individuals (Salomons et al., 2022b).

Most robots present a low (40%) to medium (48.6%) level of realism, while realistic robots are less represented and account for 11.4% of the publications (Figure 6C). Nearly all the realistic robots are objectmorphic (87.5%) and only one publication employs a realistic humanoid robot [Nadine, a robot designed to resemble a middle-aged Caucasian woman, employed in Mishra et al. (2019)].

Concerning the robot’s role, the corpus of publications studied in this review is characterized by a majority of robots acting as a peer (50%) while the subordinate and superior/tutor roles account for 27.1% and 18.6% of the publications respectively (Figure 6D).

### 3.4 Intervention types

**FIGURE 7:**
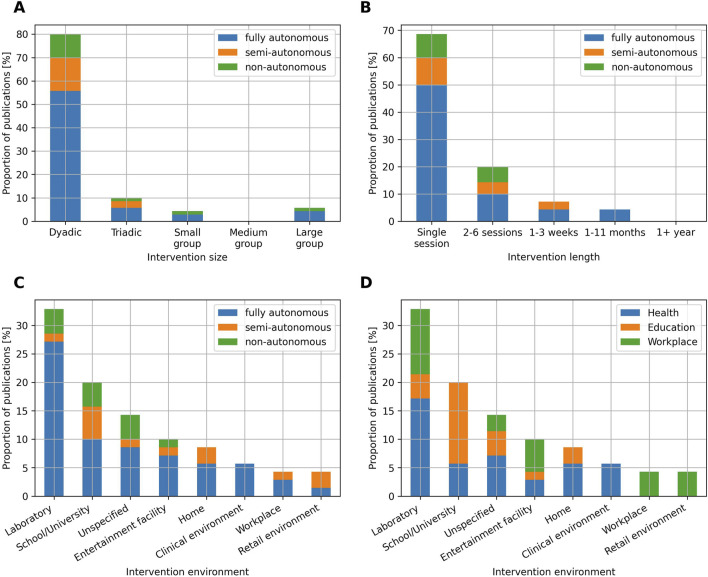
**(A)** Proportion of publications per intervention size and level of autonomy. **(B)** Proportion of publications per intervention length and level of autonomy. **(C)** Proportion of publications per intervention environment and level of autonomy. **(D)** Proportion of publications per intervention environment and field of application.

Concerning the type of intervention (Figure 7A), the vast majority of studies are dyadic (80%). Publications characterized by a triadic (Zhang et al., 2023; Kowalski et al., 2022; Charisi et al., 2021), small group (Unnikrishnan et al., 2019; Humblot-Renaux et al., 2021; Tennent et al., 2019) or large group (Velentza et al., 2021; Sackl et al., 2022) interaction sizes represent 10%, 4.3% and 5.7% respectively. No publication using a medium group intervention size is recorded. In a Kendall’s tau-b correlation analysis investigating the relationship between the interaction’s size and their representation in the publications included in this review, a strong but not statistically significant negative correlation was observed, $\tau_{b}=-0.6,\;p=0.233$ .

Most of the interactions involve only a single session (68.6%), while studies lasting 2 to 6 sessions (Chen et al., 2022; Ramachandran et al., 2019; Rajavenkatanarayanan et al., 2020), 1–3 weeks (Ramnauth et al., 2022; Mizumaru et al., 2019; Dino et al., 2019) and 1–11 months (Zhao and McEwen, 2022; Park et al., 2019; Del Duchetto et al., 2019) represent 20%, 7.1% and 4.3% respectively. No publication lasting more than 1 year is recorded (Figure 7B). In a Kendall’s tau-b correlation analysis investigating the relationship between the interaction’s length and their representation in the publications included in this review, a perfect and statistically significant negative correlation was observed, $\tau_{b}=-1,\;p=0.016$ .

Concerning the intervention environment (Figure 7C), 32.9% of the studies are conducted in a laboratory setting, while 52.9% are conducted in-the-wild, and 14.2% of the publications do not report the intervention location. The interventions performed in the real world are various: 20% of the studies are conducted in schools or universities (Kim et al., 2019; Nasir et al., 2019; Velentza et al., 2021; Charisi et al., 2021), 10% are conducted in entertainment facilities such as gym rooms (Sackl et al., 2022), museums (Del Duchetto et al., 2019; Kayukawa et al., 2023) or science festivals (Koenig et al., 2021), 8.6% are conducted at home (Ramnauth et al., 2022; Fiorini et al., 2020), 5.7% are conducted in clinical environments such as hospitals (Palinko et al., 2021), rehabilitation centres (Horn et al., 2022) or retirement homes (Odabasi et al., 2022), 4.3% are conducted in retail environments such as supermarkets (Takada et al., 2021; Lewandowski et al., 2020) or shopping malls (Mizumaru et al., 2019) and 4.3% are conducted in workplace environments such as insurance companies (Mishra et al., 2019), hotels (Nakanishi et al., 2021), *etc.* Additionally, we observe differences between the fields of applications concerning the proportion of studies conducted in the wild with respect to studies conducted in controlled-laboratory settings (Figure 7D). Education is the domain that is most tested in-the-wild, with 81.2% of the publications. On the other hand, applications related to the health and workplace domains are tested in-the-wild in 53.9% and 55.6% of the cases, respectively.

### 3.5 Levels of autonomy

**FIGURE 8:**
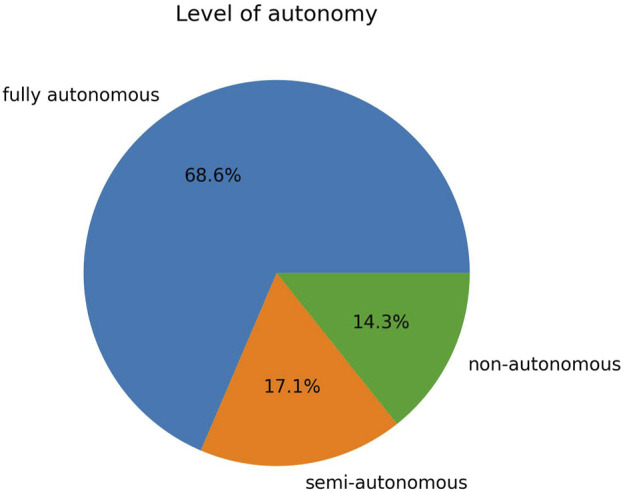
Proportion of publications per level of autonomy.

Using our search pattern, which mainly targets publications whose SAR system present some degree of autonomy, and following our coding scheme, we obtained the following distribution of level of autonomy: 68.6% of the publications employed robots able to conduct the intervention in a fully autonomous manner, 17.1% employed semi-autonomous robots, and 14.3% employed non-autonomous robots (Figure 8). We conducted statistical tests of association between the level of autonomy and the field of application, the robot’s morphology, the robot’s realism, the robot’s role, the intervention’s size, the intervention’s length and the intervention’s environment. Due to one of the assumptions of the Chi-squared test not being satisfied (expected values above five in at least 80% of the cells of the contingency table) for all these sets of variables, we used the Fisher’s exact test instead. However, due to the nature of this literature review, the row and column totals of the contingency tables could not be fixed, which is one of the assumptions of Fisher’s exact test. As a result, the tests we conducted can not be referred as exact anymore and their power is reduced. We still favoured Fisher’s exact test over alternatives such as Barnard’s test or Boschloo’s test due to the lack of available and tested software implementations (both in R and Python) of these alternative tests for more than two by two contingency tables. Note that we did not conduct a test of association between the level of autonomy and the users’ age, because each publication could fall into multiple age categories, as explained in Section 2.2. As a result, the observations are not mutually exclusive, which is another assumption of the Fisher’s exact test of association. The results of the tests we conducted are summarized in Table 1. For all the variables studied, no statistically significant associations were found (p>0.05) .

**TABLE 1:** Fisher’s exact test statistics (two-tailed).

Variable 1	Variable 2	p value
Level of autonomy	Field of application	0.336
	Robot’s morphology	0.793
	Robot’s realism	0.593
	Robot’s role	0.254
	Intervention’s size	0.660
	Intervention’s length	0.360
	Intervention’s environment	0.237

#### 3.5.1 Sensing

**FIGURE 9:**
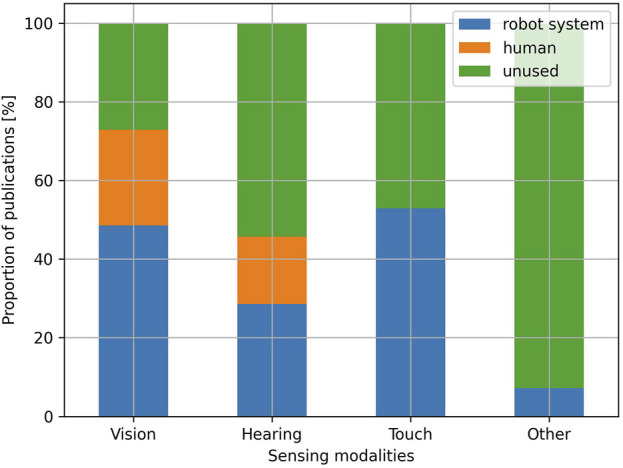
Proportion of publications per sensing modality and actor handling the modalities.

Concerning the sensing modalities (Figure 9), vision is the most prominent and is used in 72.9% of the publications. In 48.6% of the publications, the vision modality is handled fully autonomously by the robot system, while it is handled by a human in 24.3% of the publications. Publications using vision in a fully autonomous manner typically use the internal camera of the robot or external devices such as Intel RealSense or Kinect cameras. Some publications also use Lidars and laser scanners. Vision is used in a variety of ways, for example, Palinko et al. (2021); Ramnauth et al. (2022); Tozadore et al. (2022) use face/body detection to trigger specific behaviours when humans enter the robot’s field of view, Kayukawa et al. (2023); Odabasi et al. (2022) use vision to perform socially-aware navigation in real-world environments, Sackl et al. (2022); Cooney et al. (2020); Salomons et al. (2022b) use body pose estimation to provide feedback to humans during physical activities, McQuillin et al. (2022); Park et al. (2019) use vision to perform facial expression recognition, Gallenberger et al. (2019); Shaikewitz et al. (2023); Lambrecht and Nimpsch (2019) use the eye-in-hand paradigm to allow robotic arms to detect and grasp objects in their reachable workspace. This sensing modality is used for similar purposes when it is handled by humans, for example, Unnikrishnan et al. (2019); Mizumaru et al. (2019) use vision to trigger specific robot behaviours when humans enter the scene, Fiorini et al. (2020); Kouvoutsakis et al. (2022); Yadollahi et al. (2022); Gunson et al. (2022) use vision to navigate the robot in its environment, while Koenig et al. (2021) use vision to perform grasping operations.

Hearing is used to a lesser extent than vision and is present in 45.7% of the publications, with 28.6% letting hearing being handled by the robot system, and 17.1% by a human. Some publications included in this review justify not using the hearing modality with the inaccuracies of the available speech recognition solutions (Vogt et al., 2019; Donnermann et al., 2020; Tolksdorf and Rohlfing, 2020). In the cases where hearing is implemented in an autonomous manner, the internal robot microphone is typically used. External microphones such as the Amazon Echo Dot, the ReSpeaker 4-microphone array or the microphones present in Kinect devices are also used. The hearing modality is used with varying levels of complexity, for example, Ramnauth et al. (2022) uses voice activity detection: the behaviour of the robot changes according to the presence or absence of an answer from the human user, but the content of the user’s answer, if given, has no influence on the interaction. Most of the other publications adapt the interaction based on the content of the user’s answers. While some only accept a limited set of answers such as basic affirmations or negations like “yes,” “understood,” “no” (Palinko et al., 2021; Luperto et al., 2019), or basic commands like “next,” “start,” “stop,” “help” (Cooney et al., 2020; Yamamoto et al., 2021; Lambrecht and Nimpsch, 2019), others combine speech recognition with more advanced natural language understanding and dialogue management solutions (Kraus et al., 2022; Horn et al., 2022; Dino et al., 2019; Mishra et al., 2019). Finally, only a few studies extract features from the raw audio signals. For example, in Carpio et al. (2019), the robot extracts the spectrogram of the user’s audio signals and uses this information to select appropriate actions in the context of an intervention for individuals with ASD. Similarly, in Humblot-Renaux et al. (2021), the robot extracts the users’ spectrograms and gammatonegrams to perform speaker recognition. Alongside speaker recognition, they also use an array of microphones to perform sound localization, allowing the robot to align toward the current human interlocutor. Sound localization is also used in Tennent et al. (2019) to allow the robot to align toward the interlocutor who talked the least in a group. When hearing is handled by human operators, it is essentially used to perform perfect speech recognition, which is then used to react appropriately to the target user’s prompts and control the flow of the interaction. Unlike vision, where autonomous solutions seem capable to handle the same tasks occasionally delegated to humans, with comparable accuracy, automated hearing solutions seem still far from human performance, particularly in the case of speech recognition.

The touch modality is present in 52.9% of the publications, and is always handled autonomously by the robot. In most cases, this modality is employed through the use of tablets, which are often external to the robot (Apple or Android based tablets, Microsoft Surface, Wacom tablets, etc.). For example, in Vogt et al. (2019) a Microsoft Surface tablet is used as the main support for a language learning activity with children, in Kim et al. (2019) children practice handwriting on a Wacom tablet, in Kraus et al. (2022) an Android-based tablet is used to give tasks to a home assistant robot. The touch modality is also characterized by the use of force sensors, mainly by robotic arms performing grasping tasks (Gallenberger et al., 2019; Shaikewitz et al., 2023), or the use of buttons, which are often used as a way to communicate with the robot (Ramnauth et al., 2022; Kayukawa et al., 2023), or as emergency stop buttons (Odabasi et al., 2022; Dennler et al., 2021).

Finally, 7.1% of the publications used other sensing modalities. Among them, inertial sensing (Zhao and McEwen, 2022; Mizumaru et al., 2019) and heart rate/ECG sensing (Kothig et al., 2021; Farrall et al., 2023) are the most common. In Zhao and McEwen (2022), the robot possesses an inertial measurement unit (IMU) which allows detecting the child’s manipulation of the robot and triggering specific robot behaviours. In Mizumaru et al. (2019), the robot collects IMU data to perform Simultaneous Localization And Mapping (SLAM) and navigate in its environment. In Kothig et al. (2021), the heart rate of the participant is measured to adapt in real-time the difficulty of cardiovascular exercises. In Farrall et al. (2023), the heart rate of the participant is measured and is replicated on a shape-changing pneumatic sphere used for relaxation and anxiety reduction.

#### 3.5.2 Actuation

**FIGURE 10:**
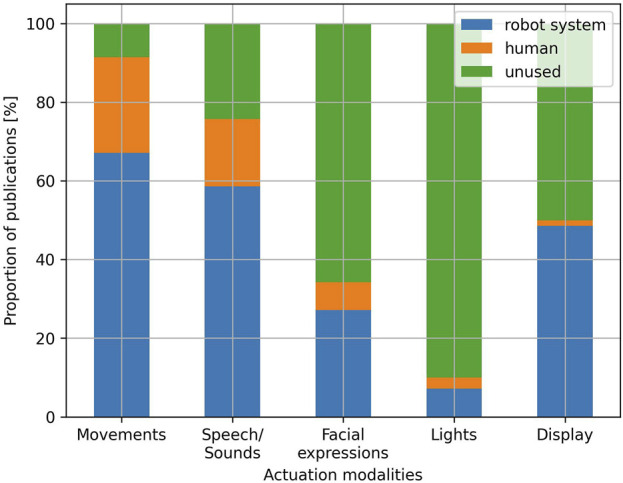
Proportion of publications per actuation modality and actor handling the modalities.

Concerning the actuation modalities (Figure 10), robot movements are the most employed and appear in 91.4% of the publications. The embodiment of robots, which is the main difference between robots and virtual agents, is thus mostly leveraged by HRI researchers. In 67.1% of the publications, the movements are handled fully autonomously by the robot system, while they are handled by a human operator in 24.3% of the publications. In the case of fully autonomous robots, movements are used in a variety of ways, for example, Kayukawa et al. (2023); Kraus et al. (2022); Odabasi et al. (2022); Del Duchetto et al. (2019) use it to navigate in the environment, Gallenberger et al. (2019); Shaikewitz et al. (2023); Salomons et al. (2022a); Lambrecht and Nimpsch (2019) use it to perform grasping and hand-over tasks, Rossi et al. (2019); Park et al. (2019) use arm and body gestures to make the robot’s emotions more expressive and realistic, Tozadore et al. (2022); Uluer et al. (2020); Dennler et al. (2021) use arm gestures to provide positive or negative feedback during child-robot interactions, Cooney et al. (2020); Kothig et al. (2021); Salomons et al. (2022b) use arm and body gestures to motivate and guide users in physical exercise interventions, Mishra et al. (2019); Nakanishi et al. (2021); Tennent et al. (2019); Humblot-Renaux et al. (2021) use it to align the robot’s head or body toward the user. Robot movements are used for similar purposes when handled by humans: in Fiorini et al. (2020); Kouvoutsakis et al. (2022); Takada et al. (2021); Nasir et al. (2019), the operators control the robots’ movements to make them navigate in the environment, in Koenig et al. (2021) the user operates the robot to perform grasping tasks, in Tolksdorf and Rohlfing (2020) a researcher operates the robot to perform pointing gestures, in Hijaz et al. (2021) a caregiver operator controls the robot’s gestures to make the robot’s emotions more expressive, in Azizi et al. (2022); Velentza et al. (2021), gestures are used to make the robot appear more lively and to complement other actuation modalities, in Tolksdorf and Rohlfing (2020) the operator controls the robot’s head to align it toward the target user.

Speech and sounds is the second most used actuation modality and is used in 75.7% of the publications, with the robot handling this modality in a fully autonomous way in 58.6% of the publications, and a human operator handling it in 17.1% of the publications. Similarly to the hearing modality, when speech is handled autonomously by the robot, it is used with varying levels of complexity. At the lowest level, speech is completely pre-programmed and does not depend on the user’s actions. For example, in Alimardani et al. (2022), the robot utterances follow a pre-programmed script, which does not depend on any sensed information from the user. Similarly, in Rossi et al. (2019), a robot uses speech to provide recommendations to the user, but these recommendations always follow the same, user-independent, structure. At the intermediary level, the flow of the interaction can typically be represented with a finite-state machine: the transition from one state of the interaction to another depends on the user’s actions, which are sensed by the robot, and each state is associated with one or more pre-programmed speech routines, keeping the overall interaction rather constrained (Luperto et al., 2019; Kim et al., 2019; Ramnauth et al., 2022; Dennler et al., 2021). Finally, no publication allowing truly open-ended conversations, such as what is permitted by the recent advancements of large language models, is recorded. This is probably mostly explained by the fact that publications using LLMs are too recent to be included in the present survey, although compliance with ethical requirements might also impact the spread of LLM-based solutions in SAR contexts. When speech is handled by a human operator, such more advanced open-ended conversation are possible: for example, in Hijaz et al. (2021), a caregiver answers children with ASD through the robot, as in normal, robot-less, therapy sessions with ASD children. Similarly, in Fiorini et al. (2020), a caregiver communicates with elderly individuals through the robot in the same way as they would normally interact with elderly people at their workplace. In most cases, however, the interaction remains constrained, with the operator taking care of managing the flow of the interaction by sending adapted pre-programmed speech routines for the robot to execute (Azizi et al., 2022; Unnikrishnan et al., 2019; Vogt et al., 2019; Chen et al., 2022). In one publication, a combination of both open-ended speech and pre-programmed speech routines is used by the operator (Nakanishi et al., 2021). While most publications use speech, sounds are also used in several publications: in Sackl et al. (2022); Kothig et al. (2021) music is used to motivate the target users during physical activity, in Farrall et al. (2023) relaxing music is used instead to help with meditation, in Antunes et al. (2022) sounds are used in an inclusive storytelling activity for children with visual disabilities while in Uluer et al. (2020) sounds are used to perform hearing rehabilitation for children with hearing disabilities. While hearing and speech are typically jointly employed in human-human interaction, it is interesting to note the gap between hearing and speech in human-robot interaction. Indeed, 43.4% of the publications using speech and sounds as an actuation modality do not use hearing as a sensing modality. Hearing, and more specifically speech recognition, suffers from several limitations which are likely to explain this gap and that will be further discussed in Section 4.4.

Concerning the use of facial expressions as an actuation modality, it is present in 34.2% of the publications, with 27.1% being handled autonomously by the robot system, and 7.1% being handled by a human operator. This modality is used is several ways, for example, Azizi et al. (2022); Kothig et al. (2021); Dennler et al. (2021) use it to display emotions and provide feedback to users, while Palinko et al. (2021); Naik et al. (2021) use it as a way to align the robot’s gaze towards the user. Many publications also simply use this modality to render the robot more lively, typically synchronizing facial expressions with speech (Dino et al., 2019; Horn et al., 2022; Dennler et al., 2021). In most publications, facial expressions are conveyed through the use of displays acting as the robot face (such as the one of QTRobot) and mainly consist of animated faces or eyes. Only a few publications use robots whose facial expressions are performed through physical mechanisms (Mishra et al., 2019; Del Duchetto et al., 2019; Unnikrishnan et al., 2019). Additionally, in a handful of publications, facial expressions consist of a human operator’s face, which is transmitted through teleoperation on the robot’s display (Fiorini et al., 2020; Bordbar et al., 2021).

Finally, the use of light as an actuation modality is pretty rare and represents only 10% of the publications included in this review. This modality is handled in a fully autonomous way by the robot in 7.1% of the publications, while it is controlled by human operators in 2.9% of the publications. It is used in a variety of ways, for example, Nakanishi et al. (2021); Rossi et al. (2019); Antunes et al. (2022) link light colours to different emotion behaviours, while Kouvoutsakis et al. (2022) uses lights to stimulate the mobility of infant children. Lights are also used to communicate robot’s states and intentions to users. For example, in Nasir et al. (2019) lights are used to communicate an imaginary battery level to children during an educational activity, in Hwang et al. (2020), lights are used to notify the user when the robot is listening, in Lewandowski et al. (2020), lights are used to display the robot’s planned trajectory.

#### 3.5.3 Transparency

**FIGURE 11:**
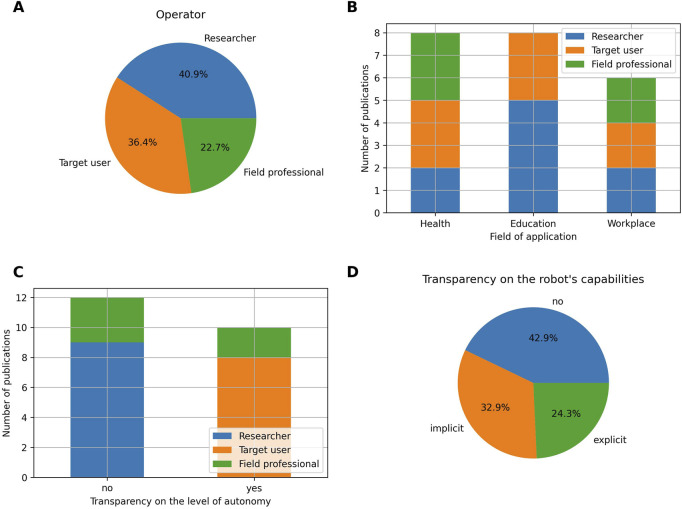
**(A)** Proportion of operators in the case of non-autonomous and semi-autonomous robots. **(B)** Number of publications with a semi-autonomous or non-autonomous level of autonomy per operator and field of application. **(C)** Number of publications per transparency on the level of autonomy and per operator. **(D)** Transparency on the robot’s capabilities.

When the robots are semi-autonomous or non-autonomous, researchers are the most common operators and represent 40.9% of the publications (Figure 11A). Researchers mainly operate robots for applications related to the field of education, but also appear in applications related to the health and workplace domains (Figure 11B). In 22.7% of the cases, the robots are controlled by field professionals. Field professionals operate robots in applications related to the health and workplace domains. No interventions related to the field of education employ field professionals as operators. It should be noted however that academic researchers were classified as researchers, although one could argue that they are also, to some extent, professionals in education. Examples of field professional operators are caregivers/therapists (Azizi et al., 2022; Fiorini et al., 2020; Hijaz et al., 2021), hotel staffs (Nakanishi et al., 2021) or policemen (Bordbar et al., 2021). Finally, robots are operated by target users themselves in 36.4% of the cases. In the field of education, the target user operators are typically children, for which controlling the robot behaviour is part of an educational activity targeting a specific learning gain. For example, in Nasir et al. (2019), the children learn about computational thinking, and more specifically path planning, by controlling the robot to go from a starting location to a goal destination while minimizing the cost of the path. Similarly, in Yadollahi et al. (2022), the children learn about perspective-taking, by controlling the robot displacements from different perspectives. In the health domain, examples of target user operators are users undergoing rehabilitation, for which controlling the robot is part of the rehabilitation process (Li X. et al., 2023), or users with disabilities for which teleoperation can help with social isolation (Mackey et al., 2022; Wang et al., 2021). Finally, in the workplace domain, Takada et al. (2021) presents a robotic shopping cart which is controlled by the target user and which performs product recommendations.

Concerning transparency, the level of autonomy is known by the user in 45.5% of the cases (Figure 11C). The level of autonomy is mainly known in applications in which the operator is the target user itself and operating the robot is part of the SAR intervention. This case accounts for 80% of the publications in which the user is aware of the level of autonomy. When researchers are the ones controlling the robot, the level of autonomy is typically not divulged to the target users. Finally, the level of autonomy is divulged in two out of five publications using field professionals as operators. In these two cases, the robot possesses a display on which the face of the teleoperator is video streamed. More specifically, in Fiorini et al. (2020), a robot is placed in the home of elderly individuals, and a caregiver teleoperates the robot to visit and chat with them on a daily basis. In Bordbar et al. (2021), a robot teleoperated by a policeman interacts with another policeman acting as a citizen.

Finally, the robot capabilities are explicitly disclosed to the users in 24.3% of the publications, implicitly disclosed in 32.85% of the publications, and not disclosed in 42.85% of the publications (Figure 11D). Examples of publication that explicitly disclose the robot capabilities are Kraus et al. (2022), in which the robot performs an introductory dialogue to the user, describing its sensory system and functionalities, or Tolksdorf and Rohlfing (2020), in which the experimenter takes care of introducing the robot in a powered-off state to children users and explains its functions: they are for example, told that the robot can talk and move by using its motors. Note that four publications included in this review employed one or several of the publication’s authors as test users of the robotic intervention (Kothig et al., 2021; Chang et al., 2022; Humblot-Renaux et al., 2021; Naik et al., 2021); we consider the robot’s capabilities to be known in such cases. Finally, publications that implicitly disclose the robot’s capabilities are publications that would not directly reveal the robot’s capabilities to the users, but would perform a warm-up session, before the official robotic intervention, to familiarize the users with the robot and the way it acts (Kayukawa et al., 2023; Dino et al., 2019; Dennler et al., 2021).

## 4 Discussion

### 4.1 Choice of population

As outlined in Section 3.2, most robotic applications are tested with early adulthood and adulthood populations, while younger and older populations are less represented. This under-representation can be attributed to several factors. First, these populations tend to be more vulnerable, which generally implies that more meticulous considerations should be made when designing robotic interventions, and more thorough ethical applications are required. These needs can be a cause for delays or cancellations of studies, which might in turn reduce the number of publications involving vulnerable participants. Similarly, the need for more carefully designed robotic interventions can be a factor leading to preliminary tests with non-vulnerable participants, and can contribute to a form of mismatch between the population targeted by the robotic interventions and the population used to test the robotic interventions. While we do not observe mismatches based on age in the publications included in this review, we notice mismatches based on other factors such as the health condition for example, (a robotic intervention designed for a population affected by a specific condition is tested on a healthy population) (Carpio et al., 2019; Cooney et al., 2020; Chang et al., 2022). Finally, the assessment of even standard HRI metrics with more vulnerable populations may also present challenges. Conventional HRI assessment means, such as questionnaires, typically do not suit well the younger populations, and alternatives, such as gamified questionnaires (Stals et al., 2024; Druga et al., 2017) or drawings (Rudenko et al., 2024) are still not widely adopted.

This review calls for more representation of the younger and older populations. These populations are not only the least represented, but are also often the most vulnerable, and therefore the ones that could potentially benefit the most from SARs. To this extent, efforts in sharing collected data (Gunes et al., 2022; Bagchi et al., 2023), as well as ensuring agreement within the community concerning which data can and should be shared, could help in developing SARs technologies for these populations and thus in reducing their under-representation. Finally, while preliminary tests on convenience populations can benefit vulnerable populations by preventing risks of harm, the results of such tests might not necessarily transfer to the vulnerable populations. As such, the review calls HRI researchers to consider such tests as a part of the iterative design process but not as a replacement of tests with the populations targeted by a robotic intervention.

### 4.2 SARs: various, humanoids and peers

As outlined in Section 3.3, the field of HRI is characterized by a plethora of different robots, with some being commercially available, and others being *ad hoc* designed. This observation can be viewed as positive since choosing or designing a robot to fit a specific intervention and target users can be key to ensuring the success of the intervention. This trend is notably enhanced by the participatory design paradigm, which aims at involving all the stakeholders in the design process of a technology to ensure that the designed technology meets the needs of its user base (Schuler and Namioka, 1993). However, the plethora of robots used can also raise difficulties. The most notable one concerns the replication of studies and their associated findings, as results that might hold for one robot might not necessarily hold for others. Furthermore, the plethora of robots used also makes transferability, and thus reusability of code more difficult, since many robots often come with their own programming interface. This review thus calls for more standardization in terms of software and hardware. Concerning the software, the use of standard middlewares such as ROS, as well as the use of robot programming conventions such as the ones presented in Mohamed and Lemaignan (2021), should be favoured whenever possible. Concerning the hardware, initiatives such as the one presented in Alves-Oliveira et al. (2022) could be further explored by the HRI community. More specifically, Alves-Oliveira et al. (2022) present a social robot embodiment kit which is intended to be flexible and to allow for customization. This kind of robot kit could represent a good tradeoff between customization, to fit a specific intervention and user base, and hardware standardization, as most of the robot hardware remains the same across different intervention cases.

As demonstrated in Section 3.3, the majority of robots employed in the field of HRI exhibit a humanoid morphology (75.7%). Because of such prevalence, researchers should remain conscious of some design principles that may negatively influence the outcomes of their intervention. One of such principles is often referred to as “Form matches function,” and states that target users expect robots to possess the sensing and actuation capabilities suggested by their appearance (Bartneck et al., 2024). In the case of humanoid robots, which are prevalent in this review, people would expect them to do human-like things such as moving, talking, hearing, seeing, *etc.* While some actuation modalities typically used by humans such as moving and talking are commonly implemented in robots (and even autonomously), as seen in Section 3.5.2, other typically human modalities, such as hearing for example, are used to a smaller extent, as described in Section 3.5.1 and further discussed in Section 4.4. As a result of such a gap, people might get disappointed and negatively perceive the robot. To prevent such issues, it might be interesting to be transparent with the user and communicate what are the actual capabilities of the robot. Alternatively, it might be worth exploring other types of robot morphology, such as zoomorphic or objectmorphic designs, which are currently underrepresented in the literature, and might even be better suited than humanoids for some contexts and applications.

The results of this review also point out that robots are predominantly programmed to act as peers during interventions, while the subordinate and tutor roles are less represented. In the field of education (Tolksdorf and Rohlfing, 2020; Park et al., 2019), justify this choice by mentioning the benefits of peer robots identified by previous research, such as the facts that children may feel more comfortable, less inhibited, and show a greater ability to focus when interacting with a robot that fulfils the role of a peer (Westlund et al., 2016; Zaga et al., 2015). Similarly (Salomons et al., 2022a), compared a robot acting as a peer with one acting as a tutor in an education context and found that participants with low prior domain knowledge learned significantly more with the peer robot. Additionally, the peer robot was also perceived as friendlier, more social, smarter, and more respectful than the tutor robot, regardless of the initial skill level of the participants (Salomons et al., 2022a). While the peer role has strong benefits, we argue that the other roles can also be successful depending on the intervention, and are worth exploring. For instance, Gargot et al. (2021) leveraged the *learning by teaching* paradigm to treat severe dysgraphia in children. More specifically, a child played the role of the tutor and the robot acted as a tutee requiring help to improve its handwriting. By doing so, positive results were achieved, such as improvements in the child’s handwriting and posture quality.

### 4.3 SARs: individualized and short-term interventions

The interventions’ size, length, and environment reflect the level of complexity of a given intervention. Large-size interventions are more challenging as they require the robotic system to have more advanced sensing capabilities, as well as take more variables into consideration in the reasoning process. Long-term interventions are challenging from a systems engineering perspective as they require the technology to be robust, intuitive, and engaging enough for repetitive use (Clabaugh and Matarić, 2019). Finally, real-world environments are more noisy and less controlled compared to laboratory settings, and thus also require more robust HRI technology (as further discussed in Section 4.4). As described in Section 3.4, the majority of studies are dyadic (80%) and only involve a single session (68.6%). These findings place the main body of SAR research on the lower end of the complexity spectrum and are consistent with the results of other reviews, such as that of Clabaugh and Matarić (2019), although their review only examines fully autonomous robots.

While focusing on dyadic interactions may reduce the overall complexity of the interventions, this also places SARs as highly individualized intervention tools, and, by extension, as a very expensive technology. It is often argued that one of the main benefits of SARs is to offer individual, adaptive and personalized interactions. While this may be appropriate for certain domains, such as health, which typically relies on individualized interactions, other domains may be affected by this viewpoint of SARs. The field of education, for instance, typically relies on group interactions, and as such, efforts in implementing SAR technology that adapts to groups instead of individuals could not only help in bridging the gap with the real world, but also in mitigating the issue of developing costly technology.

Similarly, the prevalence of single-session interventions also contributes to positioning SARs as an expensive technology, since investing significant amounts of time and effort in developing interventions with a limited lifespan is legitimately questionable. SARs have the potential (and, one could argue, the necessity) to adapt not only to higher interaction sizes but also to longer interaction lengths. This is for example, explored in Park et al. (2019), which is one of the few publications included in this review that conducts an intervention lasting more than a month. Specifically, the authors focus on children’s early literacy training through robot-mediated storytelling. The robot disposes of a large database of children’s storybooks and uses a method based on reinforcement learning to select stories that are optimized for each child’s engagement and linguistic skill progression. The prevalence of single-session interventions also highlights the fact that the main body of HRI research is subject to the novelty effect. As in the case of the intervention size, we argue that also in intervention length there is a discrepancy between the current state of HRI research and the real world. The field of education, for example, is typically a long-term process requiring practice and multiple interventions to be successful (Shute et al., 1998). Similarly, in the health domain, long-term interactions generally have a positive impact on therapeutic alliance, i.e., the collaborative relationship between a healthcare professional and a patient, which in turn, positively affects the efficacy of treatments (Martin et al., 2000). In such domains, the novelty effect greatly impacts the validity and generalizability of findings based on single-session interventions.

Other methods could be used to mitigate the issue of SARs being a costly solution. For example, efforts could be made with respect to the robot’s hardware. Three-dimensional (3D) printing and other technologies such as laser cutting or low-cost single-board computers have helped in this regard (Bartneck et al., 2024). In this review, several publications have employed robots designed with such techniques (Chang et al., 2022; Palinko et al., 2021; Unnikrishnan et al., 2019; Nasir et al., 2019; Lin et al., 2020), however, they remain a minority. This review thus suggests HRI researchers to reflect on the requirements of their research contexts, and favor technologies that are more affordable, and thus potentially more transferable to the society (such as the ones employed in the publications mentioned above or other low-cost and open-source robot designs like Flexi [Alves-Oliveira et al., 2022) or PixelBot (Maure and Bruno, 2023)], whenever their research contexts do not justify a need for expensive and advanced technologies.

### 4.4 Investigating researchers’ rationale for SARs’ levels of autonomy

In this subsection, we attempt to provide insights concerning the choice of robots’ level of autonomy at the functionality level, in an effort towards understanding the lack of researcher’s rationale identified by Elbeleidy et al. (2022).

As outlined in Section 3.5, the majority of the publications included in this review use robots that are capable of performing the intervention in a fully autonomous manner (68.6%), whereas semi-autonomous and non-autonomous robots are represented in a smaller extent, respectively accounting for 17.1% and 14.3% of the publications. The prominence of fully autonomous SARs can be partly explained by our search pattern, which mainly targets publications whose SAR system present some degree of autonomy. However, it should be noted that this prevalence was also found in the review of Elbeleidy et al. (2022), although they used a search pattern which did not enforce any specific level of autonomy, and thus represents the literature on SAR autonomy more fairly. In their review, Elbeleidy et al. (2022) mention that this vision of fully autonomous SARs by the HRI community may be partly explained by the vision of the researchers who defined the concept of SARs in the first place, who stated: “Ideally, a SAR system requires no expert operator or extensive training for use. It should be self explanatory and capable of being started, stopped, and configured by people already providing care with a minimum burden placed upon them” (Feil-Seifer and Mataric, 2005). This vision is commonly justified by stating that WoZ techniques become intractable in SAR domains requiring long-term and in-the-wild interventions (Clabaugh and Matarić, 2019).

When it comes to the other types of robot LoA (semi-autonomous and non-autonomous), this review argues that their choice over full autonomy could be motivated by the complexity of real-world environments and the limitations of the technologies typically used in the field of HRI. By observing Figure 7C for example, we notice that the majority of studies performed in a laboratory setting use fully autonomous robots (82.6%), while studies performed in the real world use fully autonomous robots to a smaller extent (62.16%). While this finding can appear counter-intuitive at first, since it can be argued that laboratory settings represent the perfect environment to control the interactions and use teleoperated robots in a semi or non-autonomous manner, it can also be argued that laboratory settings, by being more controlled, are less complex, require less robustness, and are thus simpler testbeds for fully autonomous HRI technology. To illustrate this hypothesis, we discuss the cases of two sensing modalities analysed in this review, namely, hearing and touch. To start, it is interesting to note that 71.1% of the publications not using hearing as a sensing modality use the touch modality instead. This observation is likely explained by several limitations of the hearing modality. First, hearing, and more specifically speech recognition, is affected by ambient noises. Although most speech recognition frameworks allow for ambient noise calibration, they remain a limiting factor, making speech recognition difficult to deploy, especially in non-controlled in-the-wild environments. In this review, for example, among the studies taking place in a controlled laboratory setting, hearing is handled fully autonomously by robots in 39.1% of the cases and is handled by humans in 8.69% of the cases. While keeping the predominance of robot-handled hearing, real-world settings significantly reduce this gap: hearing is handled autonomously by robots in 27% of the studies conducted in real-world settings, and by humans in 21.6% of the cases. Second, speech recognition also often suffers from timing issues. Indeed, without proper feedback on the robot side, human users often tend to speak when the robot is not yet listening. Similarly, inaccuracies in voice activity detection often result in the listening phase stopping before the end of users’ utterances. This issue can also arise when humans think and remain silent in between two utterances. Third, as mentioned in Tolksdorf and Rohlfing (2020), some populations, especially children, are often underrepresented in the training process of speech recognition techniques, which lowers the accuracy for these specific populations. In this review, only 10% of the publications involving school age and lower Erikson age categories use hearing in a fully automated way, while it is handled by a human in 30% of the cases, and not used at all in the remaining 60%. Finally, many commonly used speech recognition frameworks also face difficulties when used in interactions involving a group of individuals. For example, in this review, only 28.6% of the publications involving a triadic or bigger intervention size use hearing in a fully automated way, while the remaining 71.4% either rely on humans or do not use this modality at all. On the other hand, most of the limitations mentioned above concerning hearing do not hold for touch sensing. Indeed, the accuracy of this modality is not influenced by ambient noises nor by the age of the population using it, and it is also less impacted by timing issues and the intervention group size. Finally, while touch can allow open-ended user inputs [such as the user’s hand-writing as described in Kim et al. (2019)], it is commonly used for simple and constrained user inputs, suitable for interaction flows defined as a finite-state machine. To conclude, hearing is a good example of a sensing modality which is typically used in human-human interaction, but not used to a similar extent in human-robot interaction because of the challenges associated with it, which are yet to be overcome by the currently existing technical solutions. These technical limitations, which can also apply to other modalities, are a plausible explanation for letting a human operator handle some of the robot capabilities, or replacing them with other more robust and easily automated ones, and thus give one potential rationale for the overall choice of robots’ LoA.

As a closing remark, while some technical challenges associated to the field of HRI remain as of today, we can expect that a growing number of them will be overcome in the future. When it comes to speech recognition of underrepresented populations such as children for example, the latest models, especially the Whisper models, have shown significant improvements in the recent years (Janssens et al., 2024). Such models are also showing improvements concerning responsiveness, achieving delays below the maximum acceptable delay for human-robot interaction (≈1s) when run on local basic GPUs, and near acceptable delays for the smallest models when run on local basic CPUs (Janssens et al., 2024). Another plausible rationale for the choice of robots’ LoA lies in the robots’ operators themselves. Indeed, as seen in Section 3.5.3, and as described in the review of Elbeleidy et al. (2022), letting a human operate a robot can be desirable (and even unavoidable) when the operator is the target user and operating the robot is an integral part of the SAR intervention. In this review, such cases represent 36.4% of the publications involving human operation. Additionally, we also observe that field professionals are the least represented among human operators (22.7%). While this finding is in line with the vision of the researchers who defined the concept of SARs, stating that SARs should be self explanatory and place a minimum burden on the people already providing assistive services, this observation might also reveal feelings of fear among field professionals. Among such fears, one that is commonly mentioned is the fear of job loss and unemployment, which is particularly present in the health (Tuisku et al., 2019) and workplace domains (Wurhofer et al., 2015; Welfare et al., 2019). Such fears might induce difficulties in finding field professionals willing to test and experiment with SARs. To mitigate this issue, this review calls for more participatory and real-world research approaches, in which field professionals are actively involved in shaping the technology they are destined to use. Such research not only has the potential to boost field professionals’ understanding of the technology, but also their acceptance toward it and their confidence in using it.

### 4.5 SARs as undercover HCI

As described in Section 3.5.2, movements represent an essential aspect of SARs and are used to a great extent (91.4% of the publications included in this review). This finding highlights the desire of HRI researchers to leverage the physical embodiment of robots, which is a key element differentiating robots from virtual agents and the field of HCI. On the other hand, as mentioned in Section 3.5.2, 50% of the publications included in this review use displays as an actuation modality, and among them, 80.5% use this modality through hardware components that are external to the robot (not built-in). The fact that displays are often absent from the robots’ original design and are specifically added by HRI researchers highlights their importance in the interaction. Similarly, one can argue that the popularity of Pepper as a SAR platform, as seen in Section 3.3, is partly explained by the fact that it possesses a built-in display. In many cases, the interaction is designed and centred around the display, and the robot rather acts as an animated appendix, complementing the interaction with other actuation modalities, such as movements. While these modalities may contribute to the effectiveness of the intervention, we argue that they remain a complement to the intervention, rather than a necessity, unlike displays, without which the whole intervention would not be possible, thus revealing certain proximity of SARs interventions to HCI rather than HRI. Additionally, we argue that the use of certain modalities, such as movements in particular, solely as a complement to the interaction, underlines a sort of paradox of SARs, which was initially raised by Feil-Seifer and Mataric (2005). By constraining social robots to social gestures, and not letting them rely on direct physical interaction, researchers limit the potential of robots and their main advantage over computers and the field of HCI, which lies in their physical action capabilities. To distinguish the field of HRI from the one of HCI, researchers should reflect on whether the use of displays as the main intervention means is truly necessary, and possibly design novel human-robot interactions that truly take advantage of the robot’s physical embodiment. In that regard, we argue that assistive robots, to reach their full potential, should not be limited to social intervention only, nor to physical intervention only, but rather on a combination of the two. While a previous research workshop already introduced this concept and attempted to bring the physically assistive robotics (PAR) and the socially assistive robotics communities together, it seems that the current body of the literature does not clearly define this concept yet. As such, this review proposes the name Physically and Socially Assistive Robotic (PSAR), and calls for more collaboration between the PAR and SAR communities to provide a clear definition of PSAR as well as to advance the current state of research towards this direction.

### 4.6 Transparency

Concerning transparency with respect to the level of autonomy and the robots’ capabilities, we believe that these can have both advantages and disadvantages. First, this review argues that transparency on the robot’s capabilities can help the target users in better understanding the system they are interacting with, and thus facilitate the overall interaction. To illustrate this point, we report some of the qualitative results from a publication analyzed in this review, namely, Chen et al. (2022), in which a social robot was used in long-term in-the-wild parent-child-robot storytelling activities. More specifically, the authors report that some parent had no idea what the robot was capable of understanding and/or doing in the triadic interaction and hence had no idea how to interact with the robot properly, especially at the beginning. One of the parent who participated in their study said for example,: “[Jibo] listens to me talking about [the story] and then pipes up with a question [but] you do not really know if it is really understanding you or to what level of understanding you … .” Similarly to this lack of transparency, some of the parents commented on the lack of an initial familiarization process: “But I do admit it did take a bit of getting used to … The first time [Jibo] was talking, I found him more disruptive a bit just because I was not used to it.” While warm-up sessions are explored in some of the publications included in this review, as described in Section 3.5.3, they remain a minority, and the comments mentioned above are a testament to their importance. Finally, the comments mentioned above also highlight the importance of transparency on the robot’s capabilities, to facilitate the overall human-robot interaction.

As discussed in Section 4.2, transparency with respect to the robots’ capabilities can also benefit the interaction by reducing the risk of unmet expectations. Indeed, letting the users know what the robot is capable of, and what it is not, can be a way to avoid raising their expectations over the robots’ actual capabilities, which would in-turn help in preventing any sort of disappointment that would be detrimental to the success of the interaction (Paepcke and Takayama, 2010).

Fully autonomous SARs can also raise ethical concerns when it comes to data privacy and security (Zhong et al., 2025). Indeed, online cloud-based solutions are often leveraged to by-pass the limitations of social robots when it comes to on-board computational power. This is notably the case for some speech recognition libraries or LLMs used as dialogue manager. While these cloud-based solutions offer new opportunities for social robots, they also require users’ data to be handled by third party entities. On that regard, transparency is a necessary requirement to ensure that users are aware of the way their data is extracted and manipulated by social robots. Similarly, initiatives such as TinyML, aiming at compressing standard AI models and enabling their execution on local and low-power hardware, can also assist in reducing the reliance on cloud computing and, by extension, in safeguarding users’ data privacy. While transparency about the robots’ capabilities presents several advantages, we believe that there are specific cases in which such transparency should be avoided. If we take again the example of the Paro robot for instance, it can be argued that one of the reasons of its success resides in its design, which takes advantage of the principle “Interaction expands function.” This design principle, particularly effective for robots with limited capabilities, refers to the act of designing a robot in an open-ended way, in order to incite the target users to “fill in the blanks” left open by the design by themselves (Bartneck et al., 2024). As a result, target users invent their own way of interacting with the robot, which in turn helps in limiting disappointments linked to the robot’s limited capabilities. In such cases, transparency on the robot’s capabilities would work against such design principle, and thus limit the effectiveness of robots relying on it.

Finally, as mentioned by Riek (2012), the WoZ method raises ethical concerns, both for the target user, who is subject to deception, and for the operator, who is required to perform deception. While being transparent on robots’ LoA could represent an ethical solution, it will not change the fact that the robots are controlled by a human, nor guarantee that users will correctly understand or believe the explanation (Nasir et al., 2022).

## 5 Conclusion

This article reports a systematic review on socially assistive robotics. The review follows the PRISMA method and analyzes 70 publications published over the last 5 years. The publications are studied under different lenses: the application domains, the populations used to test the interventions, the robots’ morphology, realism and role, the interventions’ size, length and environment, the level of autonomy employed in robots based on an analysis of each of their sensing and actuation capabilities, the operators typically involved in the control of non- and semi-autonomous robots, and the transparency with respect to the robots’ level of autonomy and capabilities.

In terms of populations, the main body of SAR research focuses on adult and early adult populations, while younger and older populations are less represented and represent an opportunity for future research. The robots employed to fulfill the role of SAR are various. While this can be advantageous to select a robot that is adapted to a specific intervention and population, it also raises difficulties in terms of transferability of findings between robots, as well as transferability and reuse of code bases. To this end, more standardization is desirable and could be further explored in future research. Additionally, the vast majority of SARs exhibit a humanoid morphology, which might lead the target users to have high expectations about the robots’ capabilities, and in turn cause disappointment. On that regard, other types of robot morphology might be worth exploring in the future. Concerning the interventions in which SARs are employed, most are characterized by individualized and short-term interactions, which contribute to position SARs as an expensive technology with limited impact. Additionally, the short-term nature of SARs studies makes them prone to the novelty effect, which limits the validity of their results. Efforts should be made by the SAR research community to conduct higher-size and longer-term interventions to limit the aforementioned issues. With respect to the level of autonomy, the majority of studies we reviewed employ fully autonomous robots. While this is partly due to our search pattern, which focusses on publications whose SAR system present some degree of autonomy, the prominence of fully autonomous robots was also observed in other literature reviews, mainly the one of Elbeleidy et al. (2022), although using a search pattern representing the spectrum of SAR autonomy more fairly, and can thus be considered a real characteristic of the field. We argue that this prominence is mainly influenced by an initial vision promoting full autonomy in SAR to ensure their tractability and adoption in the real world. On the other hand, among the factors that could explain a preference for non- or semi-autonomous robots, we list intervention designs for which operating the robot is part of the assistive intervention itself, the reluctance of field professionals to use a technology that could potentially replace them, and, above all, the limitations of the current technologies required by SARs. As a result of these limitations, we point out that SARs over-rely on technology traditionally associated to HCI, such as displays and tablets, and we argue that future research, aiming to fully leverage the unique characteristics of HRI, should be dedicated to Physically and Socially Assistive Robots (PSAR): a class of robots not only relying on social interaction to provide assistance, but also taking full advantage of their embodiment, and viewing physical assistance as (also) a type of social assistance or anyway eliciting social reactions that the robot should not ignore. Finally, with respect to transparency about robots’ level of autonomy and capabilities, we argue that these can present several advantages, such as helping the target users in better understanding the system they are interacting with and thus facilitating the overall interaction, or helping in limiting disappointments that would result from over-expectations of the robots’ capabilities. However, we also identify cases in which transparency could be detrimental, and argue that the impact of transparency is still unclear, and requires further research by the community.

This review provides an analysis of socially assistive robotics centered around their level of automation. Several gaps and limitations of the current state of SAR research are identified and significant advancements in the field can be made by addressing these gaps in the future.

## Data Availability

The original contributions presented in the study are publicly available. This data can be found here: https://docs.google.com/spreadsheets/d/1u5dONx1YIGruVeWI9WjGM2jN8EUnffBZhfhXlbh6H5o/edit?usp=sharing.

## Footnotes

1 The list of the 70 included publications and their classification on the basis of the proposed coding scheme is accessible at the following link: https://docs.google.com/spreadsheets/d/1u5dONx1YIGruVeWI9WjGM2jN8EUnffBZhfhXlbh6H5o/edit?usp=sharing

2 Fast or speed reading is a technique aimed at increasing the rate at which a person reads text while maintaining comprehension. It often involves a stronger reliance on peripheral vision, with the eyes move from one line to another along a broken path in the form of a snake, rather than following each line from the beginning to the end. More details about speed reading can be found at the following link: https://en.wikipedia.org/wiki/Speed_reading.
